# Dural tear from diagnostic lumbar puncture followed by long-term morbidity: a case report

**DOI:** 10.1186/s42466-020-00083-z

**Published:** 2020-10-08

**Authors:** Aleksander Fjeld Haugstvedt, Inger Birgitte Havsteen, Hanne Christensen

**Affiliations:** 1 Department of Anaesthesia and Intensive Care, Bispebjerg and Frederiksberg Hospital, Bispebjerg Bakke 23, 2400 Copenhagen NV, Denmark; 2 Department of Radiology, Bispebjerg and Frederiksberg Hospital, Bispebjerg Bakke 23, 2400 Copenhagen NV, Denmark; 3 Department of Neurology, Bispebjerg and Frederiksberg Hospital, Bispebjerg Bakke 23, 2400 Copenhagen NV, Denmark; 4 Department of Clinical Medicine, University of Copenhagen, Blegdamsvej 3B, 2200 Copenhagen N, Denmark

**Keywords:** Spinal puncture, Post dural-puncture headache, General neurology

## Abstract

**Background:**

Lumbar punctures are performed in different medical settings and are a key procedure in the diagnosis of several neurological conditions. Complications are rare and generally self-limiting. There are no reports of symptomatic accumulation of fluid in the epidural space after lumbar puncture in adults and there are no studies on long-term outcome after post dural puncture headache (PDPH).

**Case:**

A lumbar puncture was performed in a 29 y.o. slender woman with unspecific symptoms to rule out neuro-infection. Next day MRI showed substantial accumulation of CSF in the epidural space from C2 to the sacrum dislocating the spinal chord in the spinal canal. The condition was ameliorated by epidural blood-patching. At 5 months she was still impaired by severe orthostatic headache.

**Conclusions:**

The only plausible explanation for the massive CSF leak was a dural tear occurring during multiple attempts of lumbar puncture. Anterior dislocation of the spinal chord due to CSF leak is not a recognised complication to lumbar puncture. This complication was followed by long-term disability in our case. The diagnosis can be made by MRI. A difficult procedure with several attempts and use of traumatic technique may increase risk of this complication.

Lumbar puncture is a common diagnostic procedure in neurology, as well as emergency medicine and internal medicine. Post-dural puncture headache (PDPH) is a common complication; the drawn volume of cerebrospinal fluid (CSF) correlates with PDPH frequency [1]. Serious complications including infections and hematomas are considered rare [2].

PDPH is a clinical diagnosis based on a history of recent dural puncture, either intentional or accidental, and the presence of orthostatic headache. Diagnostic criteria for PDPH have been suggested [3]. The diagnosis requires no blood samples or radiological imaging. Bed rest, fluid therapy, caffeine, analgesics, and application of epidural blood patch are used to ameliorate symptoms, which are expected to be short lasting (few days). Finally, surgical exploration and closure of the dural defect can be considered if symptoms are persistent [4–6]. Generally, evidence regarding treatment of PDPH is weak and patients are rarely seen by specialists. Epidural infections and hematomas present with symptoms raging from local back pain, through motor weakness and sensory deficit to paralysis. MRI of the spine can in most cases confirm the diagnosis [7, 8].

Symptomatic epidural collections was reported in one pediatric population [9], and further we have identified one adult case report describing severe PDPH and CSF-leak after epidural anesthesia, but in this case a distant unrelated dural leak was identified and assumed causal [10].

A 29-year-old slender female with no medical history presented to the emergency department with dizziness and unspecific pain/sensory disturbance in her lower limbs, which had developed during 5 days. She had two-year-old twins and had not been eating, drinking and sleeping properly for some days; no history of fever, nausea or vomiting.

All vital signs were normal. Somatic and neurological examinations as well as blood samples were normal including no signs of infection. A spinal tap was decided to rule out viral meningitis or other neuro-infection. The procedure was described as ‘technically challenging’ and, after several attempts, performed successfully with a traumatic 25 G needle at the level of L3/L4. CSF was normal with no signs of pleocytosis and the patient was discharged.

The patient presented again the next day with severe orthostatic headache, back pain, nausea and vomiting. The patient’s back was marked by several needle lesions and a discreet subcutaneous hematoma. Neurological examination and blood tests were normal. Because of the severity of the symptoms, especially severe pain in the back extending along the entire spine, magnetic resonance imaging (MRI) of the spine and medulla was performed 24 h after the spinal tap to exclude a spinal epidural hematoma. Substantial accumulation of liquid in the epidural space from C2 to the sacrum was identified (Fig. 1). The liquid was primarily localized posteriorly, but also laterally and anteriorly, to the dural sack, pushing the dural sac and the spinal cord anteriorly in the spinal canal and surrounding the nerve roots (Fig. 2). Due to a high-intensity signal on the T2-weighted images and a low-intensity signal on the T1-weighted images on the MRI, the liquid was interpreted as CSF rather than blood.

**Fig. 1:**
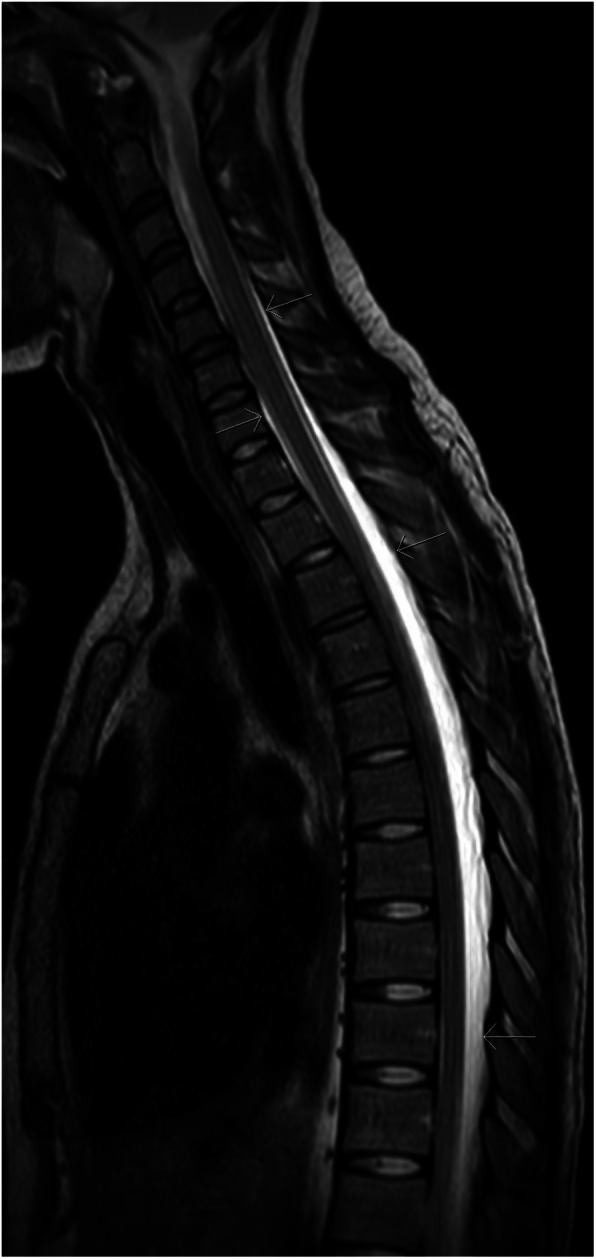
MRI (T2-weighted), upper spine, sagittal: Accumulated CSF (bright)

**Fig. 2:**
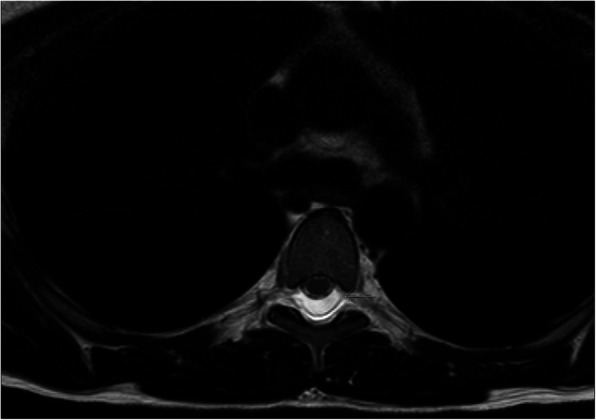
MRI (T2-weighted), TH5/TH6, horisontal: CSF surrounding nerve roots and dislocating the dural sac

The patient was treated symptomatically with pain relievers, antiemetics, plenty of fluids, caffeine and bed rest. The patient had immediate symptom relief on application of an epidural blood patch at the level of L3/L4, but the symptoms returned fully after less than 12 h. Epidural blood patch was repeated twice - after 24 and 48 h, respectively. The third epidural blood patch was persistently effective. The patient was discharged after 7 days of hospitalization.

Five months after discharge, the patient still suffered from severe headache with orthostatic aggravation, which was worsened by any physical activity. She was challenged caring for her twins and on sick-leave, unable to perform paid work.

This case documents that dural tear from lumbar puncture may cause severe morbidity presenting as a chronic and disabling PDPH. The MRI presentation with anterior dislocation of almost the entire dural sac by CSF leak into the posterior epidural space is not a recognized complication to lumbar puncture and only one adult case was found in literature. However, MRI is not a standard procedure in patients with PDPH, so we speculate if this in reality is more common than expected. MRI was performed due to a worse than usual presentation and easy access, and led to a specific diagnosis but not a change in treatment strategy.

The only plausible explanation for the massive CSF leak was a dural tear occurring during multiple attempts of lumbar puncture. In the clarity of hindsight, this lumbar puncture was not strongly indicated based on clinical findings, however, a high focus on timely diagnosis of meningococcal meningitis have led to lowering the bar for lumbar puncture in emergency care.

In conclusion, lumbar puncture may cause symptomatic dural tears leading to long-term morbidity, which underlines the importance of seeking expert help when procedures are challenging. MRI is useful in detecting CSF leak.

## Data Availability

Not applicable.
